# Closing a Backdoor to Dual Citizenship: The German Citizenship Law Reform of 2000 and the Abolishment of the “Domestic Clause”

**DOI:** 10.3389/fsoc.2020.536940

**Published:** 2020-12-15

**Authors:** Swantje Falcke, Maarten Vink

**Affiliations:** ^1^Political Science, Faculty of Arts and Social Science, University of Maastricht, Maastricht, Netherlands; ^2^Robert Schuman Centre for Advanced Studies, European University Institute, Florence, Italy

**Keywords:** naturalization, immigrants, Germany, dual citizenship, difference-in-differences analysis

## Abstract

The German citizenship law underwent a paradigmatic amendment in 2000. One often overlooked change of this reform was the abolishment of the domestic clause (“Inlandsklausel”) that implied a substantial restriction to de facto dual citizenship acceptance. Combining data from the German Socio-Economic Panel Study (waves 1993–2006) with original data on origin country policies on dual citizenship and citizenship reacquisition, we analyse the impact of the abolishment of the domestic clause on naturalization rates. We apply a difference-in-difference design to investigate the causal impact of this element of the reform which has remained under-studied. We do not find an impact of the abolishment of the domestic clause on naturalization rates, neither among the general migrant population, nor among Turkish migrants who are alleged to be targeted specifically by this reform. These results suggest that a more restrictive approach to dual citizenship did not dissuade migrants from acquiring German citizenship after 2000.

## Introduction

Over the last decades immigrants' naturalization propensity has been of growing interest to academics as well as politicians. Citizenship acquisition of immigrants is increasingly viewed as a key element to foster immigrant integration. Studies on citizenship have traditionally identified several individual and origin-country factors which determine the propensity to naturalize (Jasso and Rosenzweig, 1986; Yang, 1994; Bueker, 2005; Chiswick and Miller, 2009). Research concerning origin-country factors has looked at the relevance of institutional context, such as dual citizenship regulations at the origin-country level (e.g., Jones-Correa, 2001) as well as general accessibility of citizenship (Dronkers and Vink, 2012; Vink et al., 2013).

One of the most complex reforms of recent times is the reform of German citizenship law that came into force on 1 January 2000. Now nearly two decades ago, there are contrasting interpretations as to how and why the 2000 reform—which is generally viewed as a paradigmatic liberalization—has affected immigrant naturalization rates. Whereas, some have observed the surprising puzzle of Germany's low post-reform naturalization rates (Hochman, 2011, p. 1404; Howard, 2008, pp. 55–57; Street, 2014, p. 264) and have even concluded “that the 2000 law has been a disappointment in quantitative terms” (Green, 2012, p. 182), others have concluded that especially the reduced waiting period has increased naturalization propensity after 2000 substantially (Gathmann and Keller, 2018, p. 17).

The reform of German citizenship law in 2000 comprised of various elements. On the one hand, the reform included a major liberalizing element as it reduced the residency requirement from 15 to 8 years. Additionally, it facilitated dual citizenship for some groups. At the same time, the reform included other changes that could have negatively affected naturalization propensities. The reform introduced the birthright principle (ius soli), which grants children born in Germany automatic German citizenship at birth, irrespective of the citizenship of the parents, provided that at least one of the parents has resided in Germany for at least 7 years. If intergenerational motives drive naturalization, the introduction of ius soli would make it unnecessary for immigrant parent to naturalize to ensure that their children are citizens (Street, 2014).

While the reduction of the residency requirement (Gathmann and Keller, 2018) and the introduction of ius soli (Street, 2014) have been empirically investigated, one element of the 2000 reform has been overlooked: the abolishment of the “domestic clause.” This clause exempted German citizens, voluntarily acquiring another citizenship, from the automatic loss of German citizenship if they continued living in Germany (Hailbronner and Farahat, 2015). This clause previously enabled migrants to circumvent the effects of the German requirement to renounce one's other citizenship before naturalizing, by reapplying for their origin country citizenship after acquiring German citizenship. While the abolishment of the domestic clause has been observed by legal commentators (Hailbronner and Farahat, 2015, p. 18) and in media reports (see e.g., even recently, Middle East Monitor, 2020), it has been overlooked in all studies we are aware of that refer to aggregate naturalization statistics (Howard, 2008; Green, 2012) or analyse micro-level statistics on naturalization propensity (Hochman, 2011; Street, 2014; Gathmann and Keller, 2018)^1^.

We combine data from the German-Socio-Economic Panel Study with a unique data set on the citizenship reacquisition policies in the origin countries to investigate the impact of the abolishment of the domestic clause on naturalization rates in Germany between 1993 and 2006. Employing a difference-in-difference (DiD) strategy we, contrasting to the assumption of legal commentators, do not find an impact of the abolishment of the domestic clause naturalization rates, neither among the general migrant population, nor among Turkish migrants in particular.

The remainder of the paper is organized as follows: the next section provides a general outline of the German naturalization law reform of 2000. In the third section we provide a detailed outline of the implications of the abolishment of the domestic clause as well as an overview on existing research on naturalization propensities in Germany. This is followed by a description of the data sets used in the paper and the DiD model with which we estimate the impact of the abolishment of the domestic clause on naturalization propensities. In section Analysis, we are discussing the main results and robustness checks and provide results for an alternative specification where we focus on the effect for Turkish migrants. We end the paper with conclusions in section Conclusion.

## The German Citizenship Law Reform of 2000

For a long time, Germany was seen as a paradigmatic example of community of descent that typically was exclusive toward resident non-nationals while being inclusive toward non-resident co-ethnics (Brubaker, 1990; Green, 2012). For much of the 20th century the acquisition and loss of Germany citizenship was regulated by the Nationality Law of 1913. Since 1991 naturalization was facilitated if certain conditions were met and in 1993 this facilitation was formalized. Naturalization requirements differed depending on the age of the person in question. All immigrants had to renounce their previous nationality and show no criminal record. If between 16 and 23, immigrants were able to naturalize after residing in Germany for at least 8 years and having attended a German school for at least 6 years. Immigrants older than 23 could naturalize after 15 years given that they were able to earn a living.

After national elections in 1998, the Social Democrats (SPD) and the Green party formed the so-called Red-Green coalition, and quickly announced that one of its first legislative acts would be a reform of the citizenship law, including a paradigmatic introduction of ius soli in the German citizenship law (Howard, 2008). Following strong contestation of dual citizenship early 1999 (Green, 2005), the final proposal of the reform of the Nationality Act included some moderating elements regarding dual citizenship, which we will discuss below. Citizenship acquisition in Germany is regulated by the Nationality Act which came into force on 1 January 2000 (Hailbronner and Farahat, 2015). The new Nationality Act implied several changes regarding the conditions under which Germany citizenship could be acquired and lost. First, the residency requirement was reduced from 15 to 8 years for immigrants above 23^2^. Accordingly, the previous differentiation by age group regarding residency requirement was abolished.

Another element of the reform was the introduction of the birthright principle (ius soli), which meant that children of non-naturalized immigrants would receive German citizenship at birth if one of the parents resided in Germany for at least 8 years. Ius soli was thereby introduced as an option model. At the age of 18, children with dual nationality had to renounce either their German or foreign citizenship. This part of the reform included a transition period. Parents whose children were born between 1990 and 1999 could apply for German citizenship for their children under the birthright principle given they applied throughout 2000. However, this transition period was not used by many parents (Felfe and Saurer, 2014). Since 2014, the optional model has been modified and requirements have been relaxed. Children of immigrant parents who are born in Germany can now keep both citizenships if they lived in Germany for more than 8 years and acquired formal education in Germany, or alternatively went to a German school for at least 6 years.

Regulations on dual citizenship changed with the reform of 2000 concerning different aspects. Acceptance for dual citizenship increased as EU citizens and Swiss citizens were allowed to keep their EU citizenship under the condition of reciprocity of treatment (i.e., immigrants could keep their EU citizenship if the respective EU country also allows dual citizenship for a German in the same situation naturalizing). In 2007, the reciprocity of treatment condition was abolished, and dual citizenship was generally accepted for citizens from an EU country and Switzerland. Non-EU immigrants in principle must renounce their citizenship after 2000. However, some exceptions were included. Immigrants do not have to give up their foreign citizenship if this is not possible from the origin country, the conditions are deplorable, or the immigrant is a recognized refugee.

Since 2000, the German Nationality Act was subject to further changes and revisions. The Immigration Act of 2004, which came into force on 1 January 2005, introduced integration requirements. The residency requirement could be reduced from 8 to 7 years if the immigrant participated in an integration course including a language course as well as basic facts on German history and the political system. The 2007 Act, which came into force on 1 January 2008, added the passing of a naturalization test as an additional naturalization requirement. Additionally, the language requirements were formalized to language level B1. Immigrants with higher capabilities (e.g., B2) can naturalize already after 6 years.

**Figure 1:**
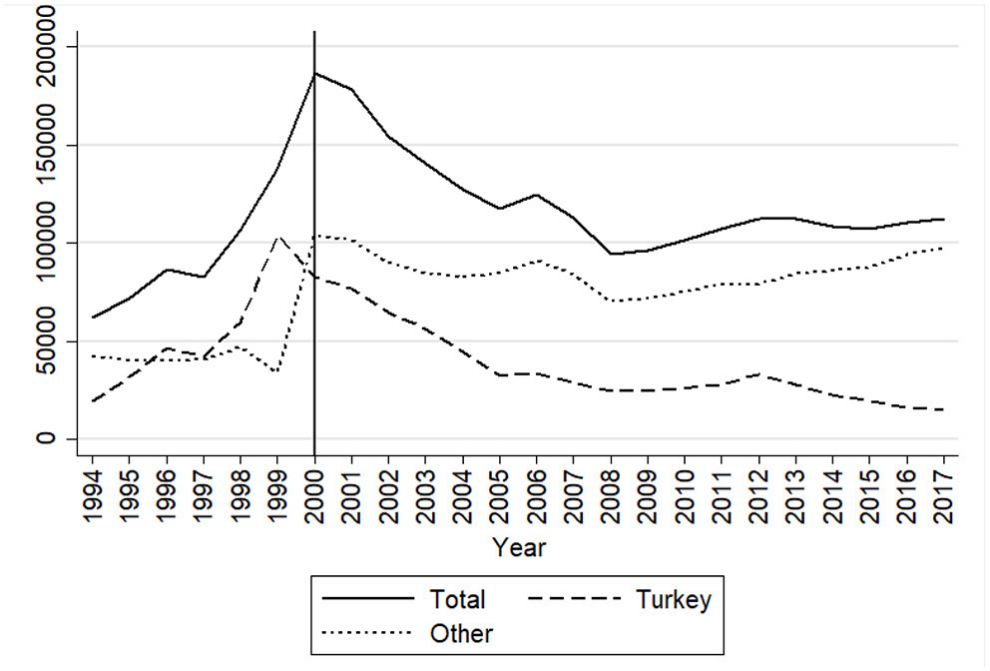
Absolute numbers of naturalisations in Germany, 1994–2017. Source: calculations by authors based on Destatis (2018). Numbers exclude ethnic Germans.

Figure 1 shows the absolute numbers of naturalizations in Germany excluding ethnic Germans since 1994^3^. Many scholars have observed the overall decreasing number of naturalizations after the reform of 2000 (in Figure 1: Total). Given the major liberalization of the reforms by reducing the residency requirement this decrease has been viewed as puzzling (Howard, 2008; Green, 2012; Street, 2014). However, the problem with such framing is that overall trends in the changing number of naturalizations ignore changes in the population that is eligible to naturalize, which are affected both by growth of the number of foreign residents as well as by the reduced residence requirement for selected groups since 1993 and generalized from 2000 onwards (as detailed above). As can be seen from Figure 1, the number of naturalizations increased since the mid-1990s, initially pushed especially by the acquisition of German citizenship by Turkish nationals (with a peak of 100,000 naturalizations in 1999) and from 2000 onwards largely driven by the non-Turkish immigrant population. In order to assess the effect of changes in the citizenship law, other than changing eligibility requirements, and net of changes in the migrant population, it is crucial to assess the rate of naturalization relative to the eligible foreign population in Germany. Unfortunately, administrative statistics on naturalization rates among the eligible population are only available since 2000 (Destatis, 2018). For this reason, analyses of the effect of the changing citizenship law in 2000, typically rely on survey data in order to estimate changes in naturalization propensity at the micro-level among migrants eligible to naturalize (e.g., Gathmann and Keller, 2018).

## Abolishment of the Domestic Clause

Despite the paradigmatic nature of the case of the 2000 German citizenship law reform, the impact on naturalization rates in Germany remains curiously understudied. In this paper we focus on one particular element of the reform that has remained under-studied so far, namely the abolishment of the so-called “domestic clause,” which concerned the closing of a previous legal loophole to circumvent Germany's overall restrictive dual citizenship policy. While this policy change has been observed by some, predominantly, legal commentators (Hailbronner and Farahat, p.18), especially in the context of the relevance of dual citizenship, no studies so far have aimed to quantify the effect of this restriction. In the next section, we introduce the context of this policy change and formulate our theoretical expectations based on the literature.

According to German law, the voluntary acquisition of another citizenship implies the automatic loss of citizenship (§ 25 StAG). Until 1 January 2000, the “domestic clause” (Inlandsklausel) allowed German citizens residing in Germany to acquire a foreign citizenship without losing the German citizenship. In practice this meant that immigrants could naturalize in Germany, give up their foreign citizenship in order to fulfill the renunciation requirement for German naturalization and re-acquire this foreign citizenship at a later stage. This practice was of particular relevance in the case of Turkish migrants who, encouraged by their home country government, made use of this circumvention of the German dual citizenship restriction at large scale (Anil, 2007; Hailbronner and Farahat, 2015). After the abolishment of the domestic clause by the reform of 2000 dual citizenship through reacquisition of the origin citizenship was no longer possible. The abolishment of the so-called “domestic clause” implied a substantial restriction to de facto dual citizenship acceptance.

First, the impact of the abolishment of the domestic clause on naturalization rates has not been investigated. This change has been addressed in legal reports (Hailbronner and Farahat, 2015), but not in those that have analyzed naturalization rates before and after 2000. The studies on the impact of the reduced residency requirement (Gathmann and Keller, 2018) and the introduction of ius soli (Street, 2014) do not explicitly account for changing dual citizenship regulations and therefore do not provide a comprehensive assessment of the various elements of the reform.

Second, there are some studies that discuss the politics of the reform, yet they refer to aggregate level statistics to formulate claims about the individual-level effects of the citizenship reform (Howard, 2008; Green, 2012; see also Anil, 2007 who provides more detailed, but still aggregate-level statistics on the naturalization practices of Turks in Germany). Other studies on naturalization propensity in Germany provide statistical analyses in a de-contextualized manner by not taking into account the effect of institutional rules in either the destination country (i.e., citizenship policy changes in Germany) or origin country (i.e., dual citizenship policies) (Diehl and Blohm, 2003; Zimmermann et al., 2009).

In the previous section, we have identified the different institutional changes due to the reform of German citizenship law in 2000. In order to carve out the effect of the abolishment of the domestic clause, it is important to identify the effect of the various changes, namely the change in residency requirement and introduction on ius soli, on the naturalization propensity of migrants.

Studies on citizenship have identified several individual and origin-country factors which determine the propensity to naturalize (Jasso and Rosenzweig, 1986; Yang, 1994; Bueker, 2005; Chiswick and Miller, 2009). These factors matter especially in terms of perceived benefits. The benefit of naturalizing depends on the relative “value” of the origin country citizenship as well as the perceived future regarding the destination country and family situation (e.g., Yang, 1994; Helgertz and Bevelander, 2017). In addition, scholars have increasingly looked at the relevance of the institutional context. The institutional context is of high importance as it shapes the naturalization process as well as the eligibility conditions. Research on this aspect has looked at the relevance of institutional context, such as dual citizenship regulations at the origin-country level (e.g., Jones-Correa, 2001) as well as general accessibility of citizenship (Dronkers and Vink, 2012). Studies including the institutional context show that restrictive policies in the destination country decrease naturalization, while more liberal policies increase naturalization (Bloemraad, 2002; Bauböck et al., 2013; Vink et al., 2013; Peters et al., 2016).

Whether or not an immigrant is eligible to naturalize is crucial when studying naturalization propensities as only then an immigrant can make the decision to naturalize. The residency requirement determines the timing of naturalization. This is important as naturalization is a life course project (Peters and Vink, 2016), and for citizenship acquisition to be part of the life planning it needs to be within a foreseeable time horizon. Thus, we expect that naturalization rates increase for all migrants after 2000 in light of the reduced residency requirement. This is supported by the results by Gathmann and Keller (2018) who do find an increased likelihood to naturalize due to the reduced residency requirement.

Migrants do not only naturalize to obtain the destination country citizenship for themselves but also for their children (Street, 2014). These intergenerational motives suggest that migrant's motivation to naturalize includes the benefits this has for their children (Street, 2014). Hence, migrants with minor children may be more likely to naturalize. This may also mean that if children can automatically acquire citizenship at birth (i.e., ius soli) migrants do not need to naturalize due to an intergenerational motive as their children will already be citizens. Thus, we expect that naturalization rates for migrants with children decreases after 2000. This decreased naturalization rates among parents has been found by Street (2014).

Naturalization propensities do not only depend on the benefits of acquisition, such as voting rights or secured residency status but also on the costs of acquisition. These costs may be monetary costs of acquiring citizenship (e.g., fees in the naturalization process) but also non-monetary costs if the origin country citizenship cannot be maintained after naturalizing. The loss of citizenship in the country of origin may affect the ability to work, hold property or invest in the origin country and can lead to a loss of rights to its public services and social benefits (Bloemraad, 2004). A general finding in the literature is that dual citizenship influences naturalization propensity (Dronkers and Vink, 2012; Peters et al., 2016)^4^. The option of dual citizenship depends on the constellation of policies in the origin (loss provision) and the destination country (renunciation requirement).

The domestic clause in Germany before 2000 implied that migrants could circumvent destination country dual citizenship restrictions by first renouncing their origin country citizenship and, subsequently, reacquiring that citizenship. Based on the costs of giving up the origin country citizenship this means that those migrants should be more likely to naturalize than their counterparts that either cannot reacquire their origin country citizenship or would subsequently lose again their destination country citizenship. Thus, we expect that naturalization rates decrease after 2000.

In the context of Germany and the domestic clause, it is known that Turkish migrants most prominently made use of the domestic clause (Hailbronner and Farahat, 2015). In German parliamentary debates regarding the abolishment of the domestic clause, this was regularly emphasized, and the abolishment was referred to as “Lex Turka” (see e.g., Deutscher Bundestag, 2005, 2008). At the same time, Turkey facilitated the use of the domestic clause by making it easy to reacquire Turkish citizenship while living in Germany (McFadden, 2019). Therefore, the abolishment of this legal loophole may have affected Turkish migrants in particular (Anil, 2007). Accordingly, naturalization rates may especially decrease for Turkish migrants after 2000.

## Material and Methodology

### Data

In order to analyse the impact of the abolishment of the domestic clause on naturalization propensities, we use information obtained from the 1993 to 2006 waves of the GSOEP^5^. The GSOEP is a longitudinal household survey, which interviews around 30,000 respondents each year. The GSOEP is the longest running longitudinal survey in Germany which enable the analysis of question on migration and integration processes (Liebau and Tucci, 2015). The survey strives to include a representative sample of migrants. Since immigrants show lower respondent rates than natives the GSOEP oversamples certain groups of immigrants—such as Turkish, Greek, Spanish, Italian, and former Yugoslavian immigrants in earlier waves (Liebau and Tucci, 2015). The GSOEP sample has been widely used to study questions of migration and integration (see on migration e.g., Diehl and Schnell, 2006; Davidov and Weick, 2011; Kóczán, 2016; and on naturalization Von Haaren-Giebel and Sandner, 2016). The GSOEP is suitable to study naturalization propensities as the questionnaire, since the beginning of the survey in 1984, includes questions about both citizenship status and country of birth.

As we are studying the effect of the abolishment of the domestic clause, we combine the GSOEP data with data sets on policies in the origin country. To test the effect of the abolishment of the domestic clause, we need to identify the group of migrants that were affected by it. This group regards migrants who are from an origin country that allows dual citizenship and provides reacquisition of citizenship without residency requirement. For information on origin country dual citizenship policies we draw on data from the MACIMIDE Expatriate Dual Citizenship Dataset (Vink et al., 2015). The information in this dataset indicates whether origin countries have policies that imply the automatic loss of citizenship upon the voluntary acquisition of another citizenship. For information on the reacquisition of citizenship, we created a new dataset—the *Reacquisition of Citizenship Dataset*—with yearly information on the possibility of citizenship reacquisition from 1960 to 2017. For the purpose of this study, we make use of a variable that differentiates between citizenship laws that (1) do not provide for the reacquisition of citizenship, (2) provide for reacquisition but with residency requirements, or (3) provides for reacquisition without residency requirement^6^.

We use the waves from 1993 as since then naturalization became formally an entitlement of individuals who fulfill the requirements (Hailbronner and Farahat, 2015, p. 4–5). Restricting the analysis to waves until 2006 enables us to have a balanced panel with 7 observation years before and from 2000. Furthermore, the selection of these survey waves allows us to exclude the impact of changes to the German citizenship law after 2006, instituting new language requirements and integration tests. The analysis focuses on first generation (i.e., foreign-born) immigrants who arrived in Germany before 1998 as they still have the possibility to naturalize within the observation period. In order to focus on the explicit decision to naturalize we restrict the analysis to immigrants who are 15 or older at the moment of migration and exclude ethnic Germans. Immigrants younger than 15 at the moment of migration can make use of different eligibility requirements as they can naturalize after having completed a minimum number of years of schooling. Furthermore, immigrants younger than 16 cannot apply for citizenship themselves but their parents apply in their name. Ethnic Germans, coming from the successor states of the former Soviet Union and from other Eastern European states are excluded as they are exempted from the standard naturalization requirements and are naturalized upon or shortly after arrival in Germany^7^. We furthermore exclude immigrant who have German citizenship at arrival^8^.

To study the impact of the abolishment of the domestic clause on naturalization rates, we restrict the sample to migrants who are eligible to naturalize. We define eligibility according to the residency requirement that an individual migrant likely faces and it lies between 3 and 15 years depending on the martial status and year of migration. Migrants who are married to a German citizen can naturalize after 3 years according to administrative practice (Hailbronner and Farahat, 2015). All other migrants face a residency requirement of 8–15 years which is determined based on their year of migration. Migrants who arrived after 2000, become eligible to naturalize after 8 years of residency. Migrants who migrated until 1985 become eligible after 15 years of residency. Migrants who arrived between 1986 and 1999 originally faced the 15 years residency requirement but given that this changes to 8 years in 2000 it was shortened to 9–14 years depending on the year of migration.

We furthermore restrict the sample to migrants who are from countries that do not automatically lose their citizenship upon naturalization in Germany but who can renounce it. The rationale for this restriction is that only migrants from countries with those dual citizenship provisions were, given the citizenship renunciation regulations in the origin country, potentially able to make use of the domestic clause. In other words, we ensure comparability between control and treatment group by restricting our sample to migrants from those origin countries with the same dual citizenship provisions. This group represents the majority of immigrants in Germany and therefore 83% of immigrants in our sample^9^.

These restrictions result in a sample of 12,147 person-year observations. As pointed out above, the GSOEP is a survey that strives to include a representative sample of migrants. The descriptive statistics of our final sample support this notion. In our sample, 39% of migrants are from Turkey, 20% from Italy and 13% from Greece. Given the sample comprises of 83% of the migrant population, these percentages are somewhat higher but corresponding with official numbers on migrant populations. For example, in 2000, the German statistical office reported that 34% of (non-naturalized) migrants in Germany were from Turkey, 10% from Italy, and 6% from Greece (Destatis, 2003).

### Estimation Strategy

We employ a difference-in-difference framework following Yasenov et al. (2019) to identify the effect of the abolishment of the domestic clause. We therefore compare naturalization rates for immigrants who were affected by the abolishment of the domestic clause (treatment) and those who were not (control) before and after 2000.

Our estimation strategy can be formalized as follows:

$$\begin{array}{l} Y_{icft}=\propto +\beta_{1}Treatment_{icft}+\beta_{2}Post_{icft}+\beta_{3}Treatment_{icft}\\ \;*{Post2000}_{t}+\beta_{4}X_{icft}+\beta_{5}O_{ct}+\gamma_{t}+\delta_{f}+\varepsilon_{icft} \end{array}$$

Where *Y*_*icft*_ indicates where an immigrant *i* from origin country *c* residing in federal state *f* is naturalized in year *t*. *Treatment*_*icft*_ indicates the treatment group and *Post*_*icft*_ indicates the years 2000 and later. *X*_*icft*_ comprises individual controls (gender, age, age-squared, years since migration, ysm-squared, years of education, marital status, citizenship spouse, child below 18, working, household income) and *O*_*ct*_ origin country controls (EU). Furthermore, the equation includes year fixed effects (γ_*t*_) to account for year-specific effects (e.g., changes in political or economic situation) as well as federal state fixed effects (δ_*f*_). ∝ denotes the intercept and ε_*icft*_ the error term^10^. We account for potential heteroskedasticity by calculating robust standard errors clustered at the individual level.

β_3_ is our difference-in-difference estimator of interest that identifies the average difference in the naturalization rate between those affected by the abolishment of the domestic clause (treatment group) and those who were not (control group) after controlling for several individual and origin country controls.

In order to identify our treatment and control group and thus who were and who were not affected by the abolishment of the domestic clause, we make use of the origin country citizenship policies and define the groups accordingly:

Treatment group: *Migrants who can reacquire their origin citizenship without residing in the origin country*.
Control group: *Migrants who cannot reacquire their origin citizenship without residing in the origin country*.

**Figure 2:**
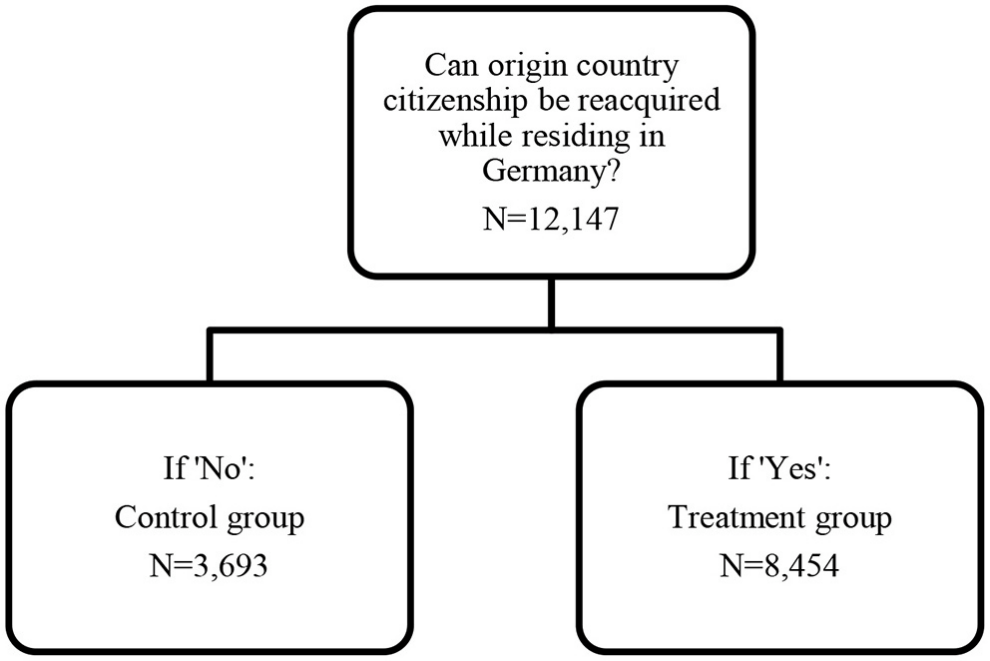
Identification of the effect of the abolishment of the domestic clause in German citizenship law.

The treatment identification is visually represented in Figure 2. Amongst migrants who are required to renounce their other citizenship, we identify whether this person would be able to reacquire this citizenship, after having renounced it, while residing in Germany. If it is possible to reacquire citizenship in the origin country without residency requirement, the migrant is in the treatment group. Otherwise, he or she is in the control group.

When analyzing the treatment effect of the domestic clause abolishment, it is important to take into account simultaneous changes that were part of the 2000 reform. As discussed in section The German Citizenship Law Reform of 2000, the reform of 2000 not only included the abolishment of the domestic clause but simultaneously reduced the residency requirement, introduced ius soli and facilitated dual citizenship for EU citizens under the condition of reciprocity of treatment. The changing residency requirement led, on the one hand to a change in the moment of eligibility but may also have affected naturalization propensities in general. Given that we restrict our analysis to eligible migrants and there are no differences in the composition between control and treatment group as well as before and after 2000 (see Supplementary Table 1), the potentially positive impact of the increased residency requirement is not affecting the results for the difference-in-difference estimator but would be reflected in general increased naturalization propensities after 2000. The ius soli introduction, which may reduce naturalization propensity of parents, does not affect our difference-in-difference estimator as the share of parents in both treatment and control group are very similar. With the reform in 2000, EU citizens and Swiss citizens were exempted from the renunciation requirement under the condition of reciprocity of treatment (i.e., immigrants could keep their EU citizenship if the respective EU country also allows dual citizenship for a German in the same situation naturalization)^11^. As a result, migrants who could keep their EU citizenship under the condition of reciprocity of treatment were not affected by the abolishment of the domestic clause. To make sure that our analysis is not biased by the inclusion of migrants from this group, we exclude migrants from these countries from the analysis in a robustness check^12^.

## Analysis

### Abolishment of the Domestic Clause

**Figure 3:**
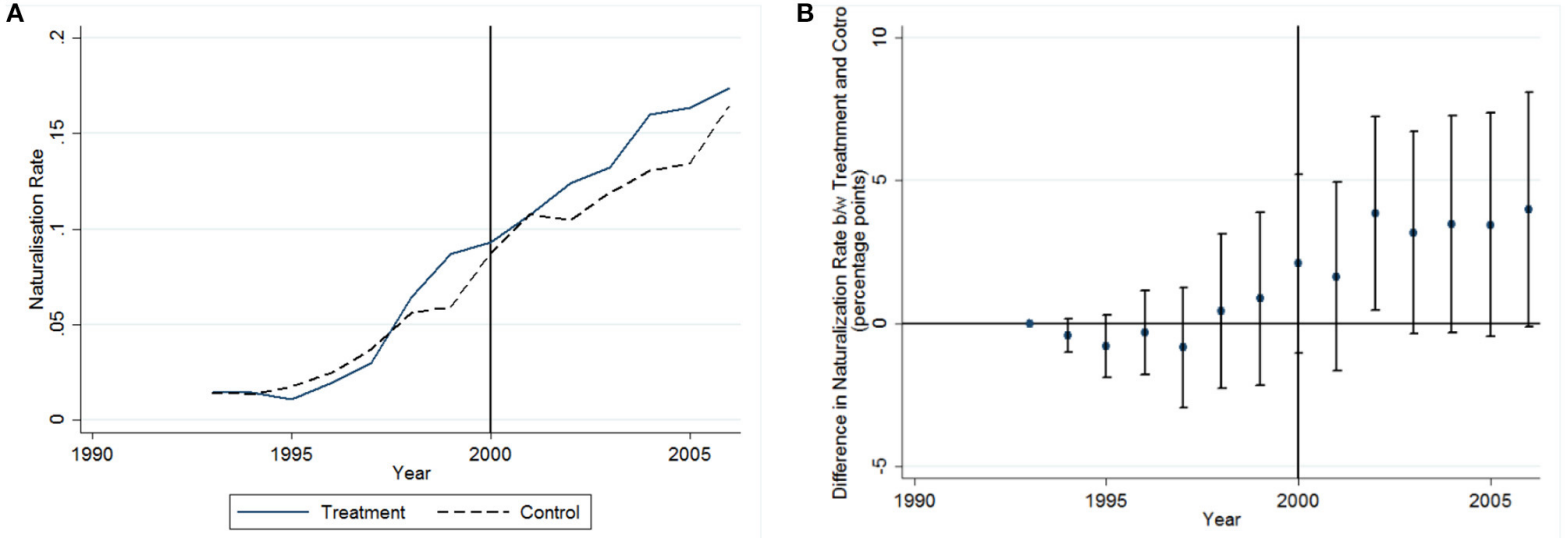
The unadjusted cumulative naturalization rate between 1993 and 2006 **(A)**, Differences in naturalization rates between Treatment and control group over time. Dots denote point estimates and vertical lines correspond to 95% CI **(B)**.

Figure 3A shows the unadjusted cumulative naturalization rates for our sample between 1993 to 2006. This naturalization rate refers to the number of migrants who are German citizens relative to the migrant residents in Germany who are eligible to naturalize^13^.

We observe continuous increasing naturalization rates within our observation window which suggests a positive effect of the formalization of the naturalization facilitation in 1993 as well as an overall positive impact of the German citizenship law reform of 2000. This suggests that there is an overall effect of the reform, but we do not observe an indication that there may be differences between control and treatment group. However, this figure is based on the raw data and in order to test for the effect of the abolishment of the domestic clause, we need to control for individual characteristics and compositional changes over time, across origin regions, and federal states within Germany.

The underlying identification assumption of the difference-in-difference design employed here is that, in absence of the abolishment of the domestic clause in 2000, naturalizations across our treatment and control groups would have followed parallel trends. Figure 3B provides evidence that the parallel trend assumption holds for our analysis. The figure presents the interaction terms of the treatment group and year indicators^14^. In order for the parallel trend assumption to hold, there should be no statistically significant difference in naturalization rates between treatment and control group before the reform of 2000. As Figure 3 shows, this is the case for our sample. However, also in the years after 2000, there is no statistically significant difference between treatment and control group indicating that the abolishment of the domestic clause may have not impacted the naturalization behavior of the treatment group.

Table 1 shows the difference-in-difference estimate of the effect of the abolishment of the domestic clause for four separate observation windows around 2000. We shorten the observation windows stepwise from 1993–2006 to 1996–2003 to do a sensitivity test of our analysis. The insignificant results of the difference-in-difference estimator in Table 1 indicate that there has been no general effect on immigrant naturalization rates of the abolishment of the domestic clause for the treatment group^15^.

**Table 1:** The impact of the abolishment of the domestic clause in 2000 on naturalization rates.

	**(1)**	**(2)**	**(3)**	**(4)**
	**1993–2006**	**1994–2005**	**1995–2004**	**1996–2003**
Difference-	0.0235	0.0242	0.0230	0.0171
in-differences	(0.0156)	(0.0152)	(0.0147)	(0.0140)
*N*	12,147	10,453	8,732	6,972

*For all coefficients, p > 0.05*.

*The outcome variables indicate whether someone is a German citizen. Results include controls for gender, age, age-squared, ysm, ysm-squared, years of education, married, married to German citizen, child below 18, working, household income, EU, year FE, federal state FE, region of origin FE. Standard errors are clustered by individuals (in parentheses)*.

### Robustness Checks

In our analyses, the control group consists of migrants who can reacquire their origin country citizenship without residing in the origin country. Thus, it consists of migrants who were affected by the abolishment of the domestic clause as, before 2000, they could be dual citizens, and afterwards not anymore. For migrants in the control group it was not possible to be dual citizens in the entire observation period. As outlined in section The German Citizenship Law Reform of 2000, the reform of 2000 included increased acceptance for dual citizenship for EU citizens and Swiss citizens under the condition of reciprocity of treatment.

Accordingly, migrants from EU countries where German immigrants could keep their citizenship, were able to be dual citizens after 2000. As this may cancel out the effect of the abolishment of the domestic clause, we exclude those countries from our analysis as a robustness check. The excluded countries are therefore: Belgium, France, Greece, Ireland, Italy, Portugal, Switzerland, UK, Sweden, Finland, Cyprus, Malta, and Slovakia. This sample is de facto a non-EU sample. In the widest window (column 1), 145 EU migrants are included who are from Romania, Poland, Hungary and Slovenia.

Table 2 shows the results for the difference-in-difference estimator for the restricted sample. Excluding migrants from our sample that can be dual citizens after 2000, does not change the results for the difference-in-difference estimator. The coefficient remains insignificant suggesting that the abolishment of the domestic clause did not affect naturalization rates among the treatment group.

**Table 2:** The impact of the abolishment of the domestic clause in 2000 on naturalization rates, excluding EU citizens that can be dual citizens after 2000 based on reciprocity.

	**(1)**	**(2)**	**(3)**	**(4)**
	**1993–2006**	**1994–2005**	**1995–2004**	**1996–2003**
Difference-in-differences	0.0277 (0.0368)	0.0345 (0.0364)	0.0395 (0.0358)	0.0347 (0.0365)
*N*	7,599	6,550	5,482	4,371

*For all coefficients, p > 0.05*.

*The outcome variables indicate whether someone is a German citizen. Results include controls for gender, age, age-squared, ysm, ysm-squared, years of education, married, married to German citizen, child below 18, working, household income, EU, year FE, federal state FE, region of origin FE. Standard errors are clustered by individuals (in parentheses)*.

### Lex Turka: The Effect for Turkish Migrants

Previous results indicate that there is no generalizable effect of the abolishment of the domestic clause. Given that Turkish migrants, encouraged by their home country government, made use of this circumvention of the German dual citizenship restriction at large scale (Anil, 2007; Hailbronner and Farahat, 2015), we want to see whether, instead of a generalizable effect, we find a particular effect among migrants from Turkey. We therefore adjust the difference-in-difference set-up comparing naturalization behavior of migrants from Turkey (treated) to migrants from other origin countries (control).

**Figure 4:**
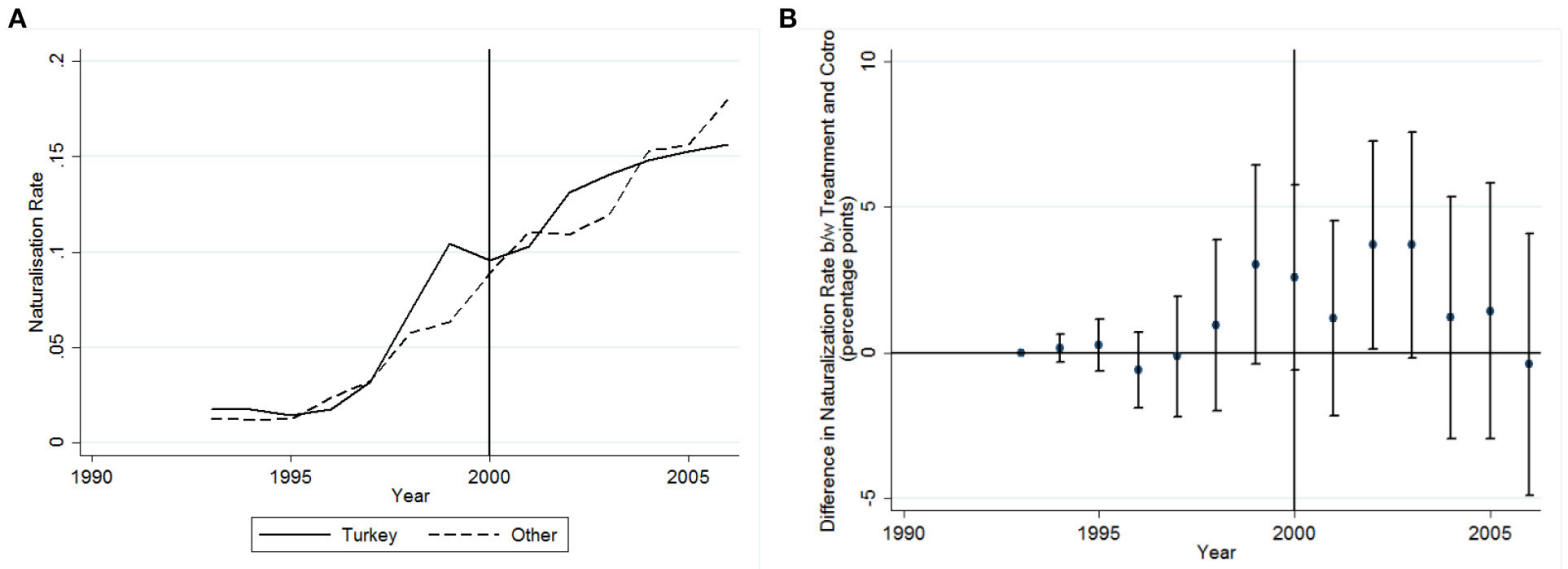
The unadjusted naturalization rate between 1993 and 2006 for Turkish vs. other migrants **(A)**, Differences in naturalization rates between Turkish migrants (treatment group) and other (control group) over time. Dots denote point estimates and vertical lines correspond to 95% CI **(B)**.

Figure 4A shows the unadjusted naturalization rates for Turkish and other migrants between 1993 to 2006. As in Figure 3, we observe increasing naturalization rates for both groups. However, the growth rate of naturalization among Turkish migrants slows down after 2000. Figure 4B shows that also for this operationalization of the difference-in-difference design the parallel trend assumption holds, meaning that there is no significant difference between treatment and control group prior to 2000.

Table 3 shows the results for the difference-in-difference estimator for Turkish migrants. The insignificant coefficient in all four observation windows suggests that there is no specific “Lex Turka” effect for Turkish migrants. Thus, the abolishment of the domestic clause did not cause decreased naturalizations among Turkish migrants.

**Table 3:** The impact of the abolishment of the domestic clause in 2000 for Turkish migrants.

	**(1)**	**(2)**	**(3)**	**(4)**
	**1993–2006**	**1994–2005**	**1995–2004**	**1996–2003**
Difference-in-differences	0.0180 (0.0149)	0.0195 (0.0143)	0.0211 (0.0137)	0.0243 (0.0131)
*N*	12,147	10,453	8,732	6,972

*For all coefficients, p > 0.05*.

*The outcome variables indicate whether someone is a German citizen. Results include controls for gender, age, age-squared, ysm, ysm-squared, years of education, married, married to German citizen, child below 18, working, household income, EU, year FE, federal state FE. Standard errors are clustered by individuals (in parentheses)*.

Hence we do not find an impact of the abolishment of the domestic clause on naturalization rates, neither among the general migrant population, nor among Turkish migrants in particular. This result is robust to samples excluding migrants who can be dual citizens based on reciprocity (Supplementary Table 3) and when comparing Turkish migrants to other migrants who were initially in the control group, thus excluding other migrants that could have been affected by the abolishment of the domestic clause (Supplementary Table 4).

How to interpret these findings? Does this mean that the pre-2000 relevance of the domestic clause has been overstated? Or, by contrast, that the possibility to circumvent dual citizenship restrictions within the context of the law of the destination country may facilitate naturalization where origin country legislation facilitates this, but once this option is off the table migrants make a new calculation. Unfortunately, our data do not allow to tease out what drives this null finding and existing research on the Turkish case points to contrasting explanations.

One the one hand, qualitative evidence from Anil (2007) suggests that dual citizenship was not the predominant issue in the naturalization decision of Turkish migrants after “the pink card system introduced by the Turkish government in 1995 removed some of the disincentives for Turkish nationals to apply for German citizenship.” (Anil, 2007, p. 1372)^16^. Others, however, question the relevance of the pink card and doubt it removed the interests of Turkish migrants to retain their Turkish citizenship:

In practice, the pink/blue card has not been as helpful as expected. Users complain that the Turkish bureaucracy was not instructed about the existence of this privileged status and so the promised advantages never materialized. In addition, this status does not protect those who might own or inherit property in military security areas, ownership remains restricted to Turkish citizens. Turks have also expressed a lack of trust in the Turkish government to continue to offer the pink/blue card. For these and other reasons, Turks who naturalized in Germany will have preferred their own particular workaround: Renunciation of Turkish citizenship, then naturalization in Germany, followed by reacquisition of Turkish citizenship and preservation of German citizenship due to the domestic exemption (McFadden, 2019, p. 78).

On the other hand, the relevance of the abolishment of the domestic clause in restricting dual citizenship is supported by a sharp decline in the number of Turks who first renounce and subsequently reacquired the Turkish citizenship from 2002 onwards. Whereas, in 2000 and 2001 on average 20,000 people reacquired Turkish citizenship per year (with a peak of 27,000 in 2001), by 2003 and 2004 the number of reacquisitions of Turkish citizenship had diminished to around 2,500 per year (Kadirbeyoglu, 2012, p. 15, Table 1). These numbers correspond with the ~48,000 Turks who have lost their German citizenship by reacquiring Turkish citizenship after 1 January 2000 (McFadden, 2019, p. 81). Such observations underline the relevance of dual citizenship for Turks in Germany, even when the abolishment of the domestic clause did not lower naturalization rates.

## Conclusion

Germany has experienced one of the most complex reforms of citizenship law in recent time with the reform of its Nationality Act in 2000. While other aspects of the reform—such as the reduced residency requirement or the introduction of ius soli—have been empirically investigated, one element has been overlooked: the abolishment of the domestic clause (“Inlandsklausel”). This paper we set out to study the impact of the abolishment of the domestic clause on naturalization rates. By doing so we aim to fill a gap between legal reports that have addressed the abolishment of the domestic clause and empirical studies on the reform of 2000 that focus on other changes.

Dual citizenship plays an important role in the naturalization decision and its option depends on the constellation of policies in the origin and destination country. The abolishment of the domestic clause implied a substantial restriction to de facto dual citizenship acceptance. The domestic clause in Germany before 2000 implied that migrants could be dual citizens by first renouncing their origin country citizenship and, subsequently, reacquire that citizenship. Thus, the abolishment may lead to a decrease in naturalization rates for migrants who, based on the origin country policies, are affected by this change. As Turkish migrants most prominently made use of the domestic clause, they may have been affected in particular.

Combining GSOEP data with data on dual citizenship and citizenship reacquisition origin country policies, we are able to study the impact of the abolishment of the domestic clause on naturalization rates of first-generation immigrants in Germany in a longitudinal manner. In this way, we follow recent studies on naturalization propensities (see e.g., Peters et al., 2016 for the Netherlands; and Helgertz and Bevelander, 2017 for Sweden), which aim to overcome shortcomings of existing cross-sectional analyses in order to identify the effect of changing institutional conditions in the destination country. In order to identify the causal impact of the abolishment of the domestic clause we employ a difference-in-difference design where we compare naturalization propensities of those migrants affected by the abolishment of the domestic clause (treatment group) to those who were not (control group).

We do not find an impact of the abolishment of the domestic clause on naturalization rates, neither among the general migrant population, nor among Turkish migrants in particular. We conclude that the abolishment of the domestic clause may have implied the closing of a backdoor to dual citizenship, by imposing a potentially severe legal consequence on the reacquisition of the citizenship of the origin country after having renounced this during the naturalization procedure, but that this apparently did not dissuade immigrants from acquiring German citizenship.

To our knowledge, we are the first to quantify the effect of this element of the 2000 citizenship law. Since, unfortunately, our data do not allow us to further probe the considerations of migrants in Germany, we invite scholars to explore the mechanisms behind these results. While there are some contrasting findings, evidence from secondary sources on balance suggest that even when the abolishment of the domestic clause did not lower naturalization rates, this does not rule out the relevance of dual citizenship for migrants in Germany.

Looking at the reform of German citizenship law in 2000, while previous research found an impact of the reduced residency requirement and the introduction of ius soli, our results indicate that a more restrictive approach to dual citizenship did not dissuade migrants from acquiring German citizenship after 2000. We thus strongly support the claim by Bloemraad (2018) that “attention to law and timing is important” when studying immigrant naturalization. States have an incentive to increase the share of naturalized immigrants, as a high share of non-nationals is a question of democratic inclusion. This is of particular relevance in Germany which shows the highest share of non-nationals (Green, 2005). The non-national population amounted to 10.6 million in 2017, of whom almost half lived in Germany for more than 15 years (Destatis, 2018). While naturalization propensity has been studied widely in other countries, research on the determinants of immigrant naturalization in Germany is still more limited than one might have expected.

Analyzing the impact of the abolishment of the domestic clause as part of the 2000 reform in Germany, has in our view, implications beyond the German case. Our paper demonstrates that it is crucial to analyse the role of dual citizenship as a constellation of origin and destination country policies.

The difference-in-difference design employed in our paper provides a robust approach to test the impact of a particular policy change, also within the context of a more complex reform including several changes as is the case with the citizenship law reform in Germany in 2000. This approach can be applied in future studies on naturalization policies in German, such as the dual citizenship liberalisations for EU citizens, or other countries.

## Data Availability Statement

The analyses in this paper are based on data from the German Socio-Economic Panel. Data access is covered by a data use agreement that does not allow us to disclose the individual-level data. Information about the data and how to access the data is available here: https://www.diw.de/en/soep. Replication material to reproduce the analyses in this paper is available at: doi: 10.7910/DVN/L3DPE4.

## Ethics Statement

Ethical review and approval was not required for the study in accordance with the local legislation and institutional requirements.

## Author Contributions

SF and MV jointly conceptualized the paper, the theoretical approach, and the empirical design. SF conducted the empirical analyses and drafted the paper. MV was responsible for the construction of the citizenship reacquisition dataset, provided continuous feedback, and revised the draft paper. Both authors have made a substantial, direct and intellectual contribution to the work, and approved it for publication.

## Conflict of Interest

The authors declare that the research was conducted in the absence of any commercial or financial relationships that could be construed as a potential conflict of interest.

## Footnotes

^1^Studies by Diehl and Blohm (2003, p. 144) and Zimmermann et al. (2009, p. 74) do not take into account the consequences of the 2000 reform due to data selection.

^2^In Germany naturalization can be acquired in three ways; with authority's discretions (Ermesseneinbürgerung), marriage to a German national, or legal entitlement (Anspruchseinbürgerung) (Green, 2012). The most common way to naturalize is through legal entitlement (Worbs, 2008). Spouses or under-aged children of Germans and naturalized immigrants can acquire German citizenship after a shorter period of residency. Refugees face a shortened residency requirement of 6 years.

^3^Since 2000, citizenship acquisition of ethnic Germans is no longer counted as naturalization and ethnic Germans are therefore no longer in the naturalization statistics. Before 2000, we proxy the number of naturalizations of ethnic Germans by excluding naturalizations via legal entitlement that are not according to par. 85 and 68 Abs.1 AuslG.

^4^See Helgertz and Bevelander (2017) for contrasting findings.

^5^Another data set that has been used to study questions on migration and integration in Germany is the Microcensus which is an annual survey of 1% of the population in Germany. While it entails a larger sample of foreigners as the GSOEP it is not suitable for our analysis as foreign-born and naturalized individuals can only be identified since 2005. Information on the year of migration and the year of naturalization could be used retrospectively but most other individual control variables cannot be recreated as this information is only available for the survey year.

^6^The dataset is publicly available at: https://doi.org/10.7910/DVN/L3DPE4. The information to construct this dataset is collected from GLOBALCIT (2017).

^7^We identify ethnic Germans by their legal status upon arrival (being ethnic German) or if the country is one of the successor states of the Soviet Union or another Eastern European state and they naturalize within 2 years after arrival.

^8^Due to small numbers of observations and data availability we exclude immigrants who report to be stateless or of Palestinian origin.

^9^The context according to which we identify which migrants do not automatically lose their citizenship upon naturalization in Germany, but who can renounce it, is laid out in section A1 in the Supplementary Material.

^10^The theoretical motivation and operationalization of the control variables is laid out in section A2 of the Supplementary Material.

^11^In 2007, the reciprocity of treatment condition was abolished and dual citizenship was generally accepted for citizens from an EU country and Switzerland.

^12^Origin countries where migrants were able to be dual citizens based on the regulations regarding EU citizenship under the condition of reciprocity of treatment are states without a generalized requirement for naturalizing persons to renounce their previous citizenship. This applies for (1) Belgium, France, Greece, Ireland, Italy, Portugal, Switzerland, and United Kingdom since 2000; (2) Sweden since 2001; (3) Finland since 2003, and (4) Cyprus, Hungary, Malta and Slovakia as new EU member states since 2004 (Vink and De Groot, 2016).

^13^Note that this naturalization rate cannot be directly compared to naturalization rates officially reported by statistical offices (such as DESTATIS and Eurostat) as these naturalization rates are calculated as the share of people acquiring citizenship in a given year relative to the foreign non-naturalization migrant population in that year.

^14^The underlying regression analysis controls for gender, age, age-squared, ysm, ysm-squared, years of education, married, married to German citizen, child below 18, working, household income, EU, year FE, federal state FE, region of origin FE. Standard errors are clustered by individuals (in parentheses).

^15^Section A4 in the Supplementary Materials provides the full Supplementary Table 2 as well as a discussion of the results for the control variables.

^16^The pink card system was introduced by the Turkish government in 1995 which enables former Turkish citizens to keep all their rights in Turkey (except the right to vote, run for public office, and work in government jobs) (Anil, 2007).
