# Supplementary material for: Closing a Backdoor to Dual Citizenship: The German Citizenship Law Reform of 2000 and the Abolishment of the “Domestic Clause”
Source: Front Sociol. 2020 Dec 15;5:536940. doi: 10.3389/fsoc.2020.536940 (PMC8022491; doi:10.3389/fsoc.2020.536940)
Supplement: Supplementary file 1 [file Table_1.docx]

**Supplementary materials**

For the paper ‘Closing a Backdoor to Dual Citizenship: The German Citizenship Law Reform of 2000 and the Abolishment of the Domestic Clause’

**A1. Renunciation requirement and dual citizenship in Germany**

In Germany, migrants who acquire citizenship are required to renounce any other citizenship they possess upon naturalisation with two main exemptions: First, if the migrant is from a country of which the citizenship legislation implies that he or she will automatically lose the origin citizenship upon naturalisation in Germany, renouncing the citizenship is not required by the German authorities. This group cannot have dual citizenship after naturalisation in Germany. We identify origin country dual citizenship acceptance based on the relevant citizenship legislation in the country of birth of a migrant. If the migrant is from a country of which the citizenship legislation implies that he or she is not allowed to voluntarily renounce the origin citizenship after naturalisation in Germany, renouncing the citizenship is not required by German authorities. This group will always have dual citizenship after naturalisation in Germany.

We identify origin country voluntary renunciation based on the relevant citizenship legislation in the country of birth of a migrant. We identify whether a country allows a person to voluntarily renounce her or his citizenship after having acquired another citizenship. Refugees, in line with Germany’s commitment under international law, are not required to renounce their other citizenship. This applied both before and after 2000. In addition, other exemptions to the renunciation requirement apply, which were somewhat broadened in 2000. Before 2000, exceptions were granted, for instance, if a foreigner could not renounce his or her previous nationality or only under particularly difficult conditions, e.g. if the ‘original home country required military service before giving up nationality’ (Farahat and Hailbronner, 2005: 5). After 2000, ‘[d]ual nationality is accepted in more cases, e.g. if the applicants are elderly persons and dual nationality is the only obstacle to naturalisation, if the dismissal of the previous nationality is related to disproportionate difficulties, and if a denial of the application for naturalisation would constitute a particular hardship; moreover, double nationality is accepted in cases in which the renunciation of the previous nationality entails – in addition to the loss of civil rights – economic or financial disadvantages.’ (Farahat and Hailbronner, 2020: 8). Due to data availability and the individualised nature of the latter exceptions, we cannot identify these on the basis of the GSOEP data.

**A2. Theoretical motivation and operationalisation of the control variables**

Studies on citizenship have identified several individual factors which determine the propensity to naturalise (Bueker, 2005; Chiswick & Miller, 2009; Jasso & Rosenzweig, 1986; Yang, 1994). In order to identify the impact of the abolishment of the domestic clause we control for these individual factors. In the following we provide the theoretical motivation to include the variable as well as the operationalisation where necessary.

*Gender*

Yang (1994) argues that for women naturalisation can be seen as a liberation from repressive marriages and occupations and women are thus more likely to naturalise than men. However, the empirical results for gender have been mixed. While Yang (1994) found this gender bias, other studies have found no effect (see e.g. Vink et al., 2013).

*Age*

Jasso and Rosenzweig (1986) argue that the older a person, the shorter the time to enjoy the benefits associated with the destination countries’ citizenship. Therefore, naturalisation propensity decreases with age. However, the empirical findings are less conclusive with Jasso and Rosenzweig (1986) finding this link, Yang (1994) finding a curvilinear relationship and Chiswick and Miller (2009) finding a positive association.

In light of these findings, we include a linear and quadratic control for age to allow for various relations.

*Years since migration*

The longer an immigrant resides in the country of destination, the more likely she or he is to acquire citizenship (Bueker, 2005). This is on the one hand due to the residency requirement but also as the commitment with the host society can be expected to increase with length of stay. This effect may decrease after a certain moment in time.

We therefore include a linear and quadratic control for years since migration.

*Years of education*

The educational level of migrants has been found to positively affect naturalisation propensities (Bevelander & Veenman, 2006; Vink et al. 2013). This may be explained by the types of jobs highly skilled migrants qualify for. If citizenship is a precondition, they are more likely to naturalise (Vink et al., 2013).

We include a variable comprising of four categories of similar size each regarding to a maximum number of years of education: (1) 7 to 9 years, (2) 10-12 years, (3) 13-15 years, (4) 16-19 years*.*

*Marital status*

Yang (1994) hypothesizes that marriage increases the propensity of immigrants to naturalise as the costs of returning to their country of origin is higher for them and families in general are in closer contact with institutions of the country of destination which makes it easier to integrate. Helgertz & Bevelander (2016) and Peters, Vink & Schmeets (2016) look at the citizenship characteristics from the spouse and find that their citizenship status matters: Being married to a citizen (native or naturalised) increases the naturalisation propensity, while being married to a non-naturalised foreign citizen, decreases the propensity to naturalise.

In order to account for the citizenship status of the partner we include a variable to capture the role of marriage in general and a variable indicating whether the spouse is a German citizen in particular.

*Child*

Concerning the presence of children, Yang (1994) as well as Street (2014) emphasize the intergenerational motivation for naturalisation. Naturalised parents make it easier for their children to naturalise. Furthermore, children may increase their immigrant parents’ intentions to stay in the country of destination. The empirical evidence on the role of children on the decision to naturalise is mixed. While Yang (1994) finds a positive effect of the presence of children on naturalisation, several other studies find no effect (see e.g. Bevelander & Veenman, 2006, Vink et al., 2013).

*Working and household income*

Both having paid work and household income are indications of the ability to naturalise of a migrant. Many countries, including Germany, have requirements for economic self-sufficiency for a migrant to be eligible to naturalise. Accordingly, having paid employment or higher household income increases naturalisation propensity.

We measure working as a binary variable that indicates where a person has paid employment or not. Household income is measured as annual household income as a categorical variable comprising of four categories: (1) Up to €15133, (2) €15133-€28120, (3) €28121-€43766, and (4) more than €43,766.

*EU*

The economic and political situation in the country of origin is influencing the propensity to naturalise (see e.g. Bueker, 2005; Peters, Vink & Schmeets, 2016). In this context migrants from the European Union will be less likely to acquire German citizenship as, due to the freedom of movement, they already enjoy many of the rights associated with German citizenship.

We use a binary variable and define EU membership dynamically indicating whether the origin country is a member of the EU in a given year.

For some of the control variables information is missing (Years of education, working and household income). For those variables we include a separate category labelled ‘(variable name) unknown’*.*

**A3. Descriptive statistics (sample 1993-2006), in percent**

Table A1 shows the summary statistics for our overall sample (first column), treatment group (second column), control group (third column), before 2000 (fourth column) and after 2000 (fifth column). The numbers of observations before and after 2000 are very balanced. Treatment and control group, as well as the observations before and after 2000, are comparable regarding individual characteristic but the control group has, by design, a higher share of EU migrants.

**Table A1.** Summary statistics

| Variable |  | Total | Treatment | Control | Pre-2000 | Post-2000 |
| --- | --- | --- | --- | --- | --- | --- |
| German citizen | Yes | 8.16 | 8.43 | 7.53 | 3.16 | 12.95 |
|  | No | 91.84 | 91.57 | 92.47 | 96.84 | 87.05 |
| Female | Yes | 47.73 | 49.55 | 43.57 | 45.38 | 49.98 |
|  | No | 52.27 | 50.45 | 56.43 | 54.62 | 50.02 |
| Age | Mean (SD) | 51.46  (11.32) | 51.29  (24.90) | 51.84  (11.43) | 51.35  (9.62) | 51.56  (12.74) |
| Ysm | Mean (SD) | 25.25  (8.98) | 24.90  (8.69) | 26.05  (9.57) | 25.11  (6.55) | 25.38  (10.81) |
| Married | Yes | 86.89 | 88.64 | 82.86 | 88.49 | 85.34 |
|  | No | 13.11 | 11.36 | 17.14 | 11.51 | 14.66 |
| Married to citizen | Yes | 18.12 | 16.49 | 21.85 | 11.40 | 24.56 |
|  | No | 81.88 | 83.51 | 78.15 | 88.60 | 75.44 |
| Working | Yes | 44.92 | 41.51 | 52.75 | 49.10 | 40.92 |
|  | No | 43.68 | 47.00 | 36.10 | 40.93 | 46.32 |
|  | Unknown | 11.39 | 11.50 | 11.16 | 9.97 | 12.75 |
| Years of education | 7-9 | 55.12 | 56.17 | 52.72 | 62.42 | 48.13 |
|  | 10-12 | 33.74 | 32.80 | 35.88 | 31.40 | 35.97 |
|  | 13-15 | 7.62 | 7.32 | 8.29 | 4.02 | 11.06 |
|  | 16-19 | 2.51 | 2.72 | 2.03 | 1.45 | 3.53 |
|  | Unknown | 1.01 | 0.98 | 1.08 | 0.71 | 1.31 |
| Household income | <15133 | 23.52 | 25.03 | 20.06 | 18.30 | 28.52 |
|  | 15133-28120 | 18.11 | 18.74 | 16.68 | 22.14 | 14.25 |
|  | 28121-43766 | 23.15 | 22.75 | 24.07 | 25.50 | 20.90 |
|  | > 43766 | 25.61 | 23.75 | 29.87 | 26.02 | 25.22 |
|  | Unknown | 9.61 | 9.74 | 9.31 | 8.04 | 11.11 |
| Child18 | Yes | 15.81 | 16.15 | 15.03 | 15.21 | 16.38 |
|  | No | 84.19 | 83.85 | 84.97 | 84.79 | 83.62 |
| EU | Yes | 37.36 | 23.37 | 69.37 | 40.49 | 34.36 |
|  | No | 62.64 | 76.63 | 30.63 | 59.51 | 65.64 |
| N |  | 12,147 | 8,454 | 3,693 | 5,945 | 6,202 |

**A4. Main difference-in-difference analysis; Full table**

***Table A2.*** *The impact of the abolishment of the domestic clause in 2000 and other personal characteristics on naturalisation rates.*

|  |  | (1) | (2) | (3) | (4) |
| --- | --- | --- | --- | --- | --- |
|  |  | 1993-2006 | 1994-2005 | 1995-2004 | 1996-2003 |
| Control/Treatment group | Control | *Ref.* | *Ref.* | *Ref.* | *Ref.* |
|  | Treatment | -0.0144 | -0.0158 | -0.0171 | -0.0176 |
|  |  | (0.0131) | (0.0136) | (0.0145) | (0.0157) |
| Difference-in-differences |  | 0.0235 | 0.0242 | 0.0230 | 0.0171 |
|  |  | (0.0156) | (0.0152) | (0.0147) | (0.0140) |
| Post2000 |  | 0.0594^**^ | 0.0558^***^ | 0.0608^***^ | 0.0442^**^ |
|  |  | (0.0183) | (0.0169) | (0.0159) | (0.0147) |
| Gender | Male | Ref. | Ref. | Ref. | Ref. |
|  | Female | 0.0208 | 0.0235 | 0.0262^*^ | 0.0277^*^ |
|  |  | (0.0125) | (0.0126) | (0.0130) | (0.0135) |
| Age |  | -0.0055 | -0.0059 | -0.0058 | -0.0062 |
|  |  | (0.0045) | (0.0047) | (0.0050) | (0.0053) |
| Age^2^ |  | 0.0000 | 0.0001 | 0.0001 | 0.0001 |
|  |  | (0.0000) | (0.0000) | (0.0000) | (0.0001) |
| Ysm |  | 0.0273^***^ | 0.0273^***^ | 0.0267^***^ | 0.0253^***^ |
|  |  | (0.0044) | (0.0047) | (0.0051) | (0.0053) |
| Ysm^2^ |  | -0.0005^***^ | -0.0005^***^ | -0.0004^***^ | -0.0004^***^ |
|  |  | (0.0001) | (0.0001) | (0.0001) | (0.0001) |
| Yeas of education | 7-9 | *Ref.* | *Ref.* | *Ref.* | *Ref.* |
|  | 10-12 | 0.0419^**^ | 0.0418^**^ | 0.0437^**^ | 0.0455^**^ |
|  |  | (0.0139) | (0.0142) | (0.0147) | (0.0152) |
|  | 13-15 | 0.0592 | 0.0601 | 0.0581 | 0.0594 |
|  |  | (0.0337) | (0.0350) | (0.0367) | (0.0389) |
|  | 16-19 | 0.124 | 0.136^*^ | 0.142^*^ | 0.158^*^ |
|  |  | (0.0659) | (0.0663) | (0.0676) | (0.0710) |
|  | Unknown | -0.0428 | -0.0427 | -0.0467 | -0.0585 |
|  |  | (0.0502) | (0.0494) | (0.0511) | (0.0477) |
| Married (yes) |  | -0.0877^***^ | -0.0841^***^ | -0.0848^***^ | -0.0881^***^ |
|  |  | (0.0169) | (0.0175) | (0.0188) | (0.0204) |
| Married to DE citizen (yes) |  | 0.336^***^ | 0.341^***^ | 0.347^***^ | 0.351^***^ |
|  |  | (0.0305) | (0.0314) | (0.0326) | (0.0340) |
| Working | No | *Ref.* | *Ref.* | *Ref.* | *Ref.* |
|  | Yes | -0.00582 | -0.00414 | -0.00110 | 0.0000755 |
|  |  | (0.0103) | (0.0108) | (0.0115) | (0.0126) |
|  | Unknown | -0.00996 | -0.00461 | -0.00870 | -0.0121 |
|  |  | (0.0255) | (0.0256) | (0.0251) | (0.0237) |
| Child < 18 (yes) |  | -0.000313 | -0.00163 | -0.00242 | -0.00406 |
|  |  | (0.0149) | (0.0154) | (0.0162) | (0.0172) |
| Household income | Until 15,133 | Ref. | Ref. | Ref. | Ref. |
|  | 15,133-28,120 | 0.0128 | 0.0144 | 0.0215 | 0.0201 |
|  |  | (0.0116) | (0.0121) | (0.0128) | (0.0140) |
|  | 28,121-43,766 | 0.000550 | -0.00109 | -0.000467 | -0.00422 |
|  |  | (0.0125) | (0.0128) | (0.0132) | (0.0145) |
|  | More than 43,766 | -0.0140 | -0.0156 | -0.0143 | -0.0187 |
|  |  | (0.0136) | (0.0142) | (0.0151) | (0.0166) |
|  | Unknown | 0.00568 | -0.00261 | 0.00392 | 0.00154 |
|  |  | (0.0266) | (0.0267) | (0.0263) | (0.0262) |
| EU (yes) |  | -0.0260 | -0.0309 | -0.0380^*^ | -0.0516^*^ |
|  |  | (0.0162) | (0.0170) | (0.0183) | (0.0206) |
| *N* |  | 12147 | 10453 | 8732 | 6972 |

^*^ *p* < 0.05, ^**^ *p* < 0.01, ^***^ *p* < 0.001

Note: The outcome variables indicate whether someone is a German citizen. Results include controls for year FE, federal state FE, region of origin FE. Standard errors are clustered by individuals (in parentheses)

The positive and significant coefficient for the post2000 dummy indicates that migrants are more likely to naturalise after 2000. This reflects the reduced residency requirement since 2000 and indicates that a foreseeable time horizon is important for the naturalisation decision of immigrants.

The results for the control variables are in line with previous research as discussed in section A2. The evidence for gender is mixed – with no effect in the wider observation windows. In the narrower observation windows, women are somewhat more likely to naturalise. We do not find an effect of age, neither linear nor curvilinear. Years since migration is a strong predictor and the results suggest a curvilinear relationship. Migrants are more likely to naturalise the longer they are in Germany, but the effect decreases after a while. Marriage is a strong predictor for propensity to naturalise. If someone is married to a German citizen this increases naturalisation propensity, while marriage with a non-naturalised foreign spouse decreases naturalisation propensity. The results show no effect of children below 18 in the household, working, or household income on naturalisation propensity. The longer an immigrant is educated, the more likely he or she is to naturalise. Relative to migrants with less than 7 to 9 years of education, this holds particularly for migrants who have 13-15 year of education and to a lesser extent for 16-19 years of education

**A5. Robustness checks Turkey**

For this approach, we define treatment and control as in section 5.3, but exclude countries where migrants could be dual citizens after 2000 based on EU reciprocity.

**Table A3.** the impact of the abolishment of the domestic clause in 2000 for Turkish migrants, excluding EU citizens that can be dual citizens after 2000 based on reciprocity.

|  | (1) | (2) | (3) | (4) |
| --- | --- | --- | --- | --- |
|  | 1993-2006 | 1994-2005 | 1995-2004 | 1996-2003 |
| Difference-in-differences | 0.0153 | 0.0188 | 0.0217 | 0.0258 |
|  | (0.0176) | (0.0170) | (0.0163) | (0.0155) |
| *N* | 8441 | 7263 | 6061 | 4832 |

^*^ *p* < 0.05, ^**^ *p* < 0.01, ^***^ *p* < 0.001

Note: The outcome variables indicate whether someone is a German citizen. Results include controls for gender, age, age-squared, ysm, ysm-squared, years of education, married, married to German citizen, child below 18, working, household income, EU, year FE, federal state FE. Standard errors are clustered by individuals (in parentheses)

In an additional robustness check (Table A4) we exclude non-Turkish migrants that were previously in the treatment group from the sample. Thus, Turkish migrants are the treatment group and the control group is the same as in the origin analysis.

**Table A4.** the impact of the abolishment of the domestic clause in 2000 for Turkish migrants, excluding other migrants affected from the abolishment of the domestic clause.

|  | (1) | (2) | (3) | (4) |
| --- | --- | --- | --- | --- |
|  | 1993-2006 | 1994-2005 | 1995-2004 | 1996-2003 |
| Difference-in-differences | -0.00834 | -0.00191 | 0.00628 | 0.0163 |
|  | (0.0214) | (0.0210) | (0.0204) | (0.0203) |
| *N* | 7599 | 6550 | 5482 | 4371 |

^*^ *p* < 0.05, ^**^ *p* < 0.01, ^***^ *p* < 0.001

Note: The outcome variables indicate whether someone is a German citizen. Results include controls for gender, age, age-squared, ysm, ysm-squared, years of education, married, married to German citizen, child below 18, working, household income, EU, year FE, federal state FE. Standard errors are clustered by individuals (in parentheses)

REFERENCES

Bevelander, P., and Veenman, J. (2006). Naturalization and employment integration of Turkish and moroccan immigrants in the Netherlands. *J. Int. Migr. Integr.* 7, 327–349 doi: 10.1007/s12134-006-1016-y
